# Invasive Aspergillosis in a Renal Transplant Recipient Successfully Treated with Interferon-Gamma

**DOI:** 10.1155/2012/493758

**Published:** 2012-09-27

**Authors:** C. Estrada, A. G. Desai, L. M. Chirch, H. Suh, R. Seidman, F. Darras, E. P. Nord

**Affiliations:** ^1^Division of Nephrology, Department of Medicine, School of Medicine, State University of New York at Stony Brook, HSC T-16 Rm 080, Stony Brook, NY 11794, USA; ^2^Division of Pulmonology, Department of Medicine, School of Medicine, State University of New York at Stony Brook, Stony Brook, NY 11794, USA; ^3^Division of Infectious Diseases, Department of Medicine, School of Medicine, State University of New York at Stony Brook, Stony Brook, NY 11794, USA; ^4^Department of Pathology, School of Medicine, State University of New York at Stony Brook, Stony Brook, NY 11794, USA; ^5^Transplantation Services, School of Medicine, State University of New York at Stony Brook, Stony Brook, NY 11794, USA

## Abstract

Invasive aspergillosis is a serious complication of solid organ transplantation. An early diagnosis is hampered by the lack of reliable serum markers and, even if appropriately diagnosed and treated with current antifungal agents, has a high mortality rate. We report a case of invasive pulmonary and cerebral aspergillosis in a renal transplant patient treated with IFN-**γ** in conjunction with combination anti-fungal therapy for six weeks in whom complete resolution of the fungal infection was achieved. Renal function remained intact throughout the treatment period. Surveillance CT scans of the chest and head showed resolution of prior disease but revealed a new left upper lobe mass four months after completion of treatment with IFN-**γ**. Biopsy of the lesion was positive for primary lung adenocarcinoma, for which she underwent left upper lobe resection. The pathology report confirmed clear surgical margins and lymph nodes and no evidence of fungal hyphae. IFN-**γ** should be considered early in the management of invasive aspergillosis in renal transplant patients. To date, allograft rejection has not been encountered.

## 1. Introduction

Invasive aspergillosis (IA) is a serious complication of solid organ transplantation. Early diagnosis improves mortality but can be challenging [1]. The introduction of voriconazole has played a role in decreasing morbidity and mortality, when compared to amphotericin B [2], but concern exists regarding mounting azole resistance [3], and mortality remains as high as 70% [4, 5].

The use of interferon-gamma (IFN-*γ*) to treat invasive fungal infections is derived from animal models that showed resistance to disease correlated with an intact Th1 response [6] and protection was associated with high levels of tumor necrosis factor alpha (TNF-*α*), interleukin-2 (IL-2), interleukin-12 (IL-12), and IFN-*γ* [7]. Current immunosuppressive therapy blunts cell-mediated immunity, thereby predisposing organ transplant recipients to invasive fungal infections. IFN-*γ* has the potential to augment this defect in immunity, eradicate invasive fungal disease, and thus far has not been associated with allograft rejection [8]. We report a case of invasive pulmonary and cerebral aspergillosis, coinfected with cytomegalovirus (CMV) pneumonitis, in a renal transplant recipient, successfully treated with adjunctive IFN-*γ*, after combination antifungal therapy failed to eradicate the infection.

## 2. Case Report

A 54-year-old woman recently diagnosed with non-small-cell lung cancer was admitted for a left upper lobe resection. Her past medical history was significant for chronic bronchitis, hepatitis C treated with interferon-alpha in 1992, and hypertension. She also had chronic kidney disease secondary to glomerulonephritis (not biopsy proven), for which she had a preemptive living-unrelated kidney transplant in November 2009. She was an active smoker. 

**Figure 1:**
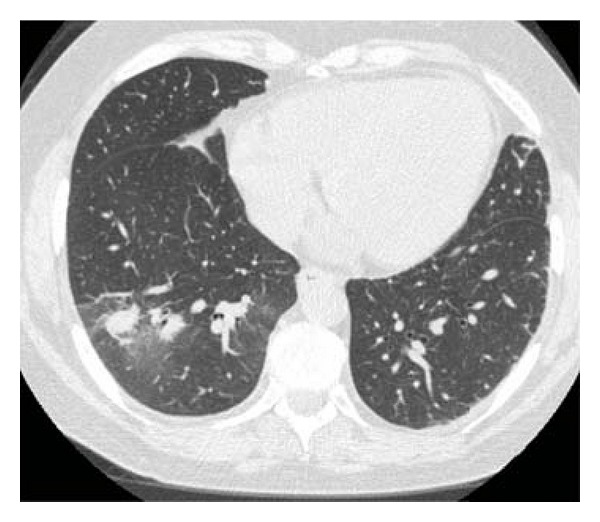
Chest CT scan without intravenous contrast on initial admission showing patchy infiltrates and a “halo sign” surrounding a right lower lobe lesion.

Nine months posttransplant, she presented to the emergency department with a cough and dyspnea for one month. On physical examination, she was afebrile with a blood pressure of 121/56 mmHg, a heart rate of 68 beats per minute and regular, and an oxygen saturation of 97% on room air. Chest examination was significant for diffuse expiratory wheezes with scattered crackles. Heart, abdomen, and neurologic examinations were normal. Her medications included tacrolimus and mycophenolate mofetil. On laboratory evaluation, she had a white blood cell count of 9.5 × 10^3^/mL^3^ with 93.8% neutrophils, blood urea nitrogen of 25 mg/dL, and a serum creatinine of 1.1 mg/dL. Serum tacrolimus level was elevated at 14.5 mcg/mL. Computed tomography (CT) scan of her chest (Figure 1) showed multifocal infiltrates and a halo sign. Aztreonam, vancomycin, metronidazole, and capsofungin were initiated on admission for presumed multifocal pneumonia. Serum cytomegalovirus (CMV) polymerase chain reaction (PCR) and galactomannan antigen were negative. Due to lack of clinical improvement, a CT guided lung biopsy was attempted, and complicated by hemopneumothorax and cardiac arrest, from which she recovered. The biopsy result was inconclusive but sputum culture subsequently revealed moderate *Aspergillus fumigatus*. Treatment for pulmonary aspergillosis was initiated with intravenous voriconazole (6 mg/kg twice daily) for one day and then changed to oral voriconazole (4 mg/kg twice daily), which she was discharged on. Tacrolimus and mycophenolate mofetil were suspended and prednisone was initiated in their place.

**Figure 2:**
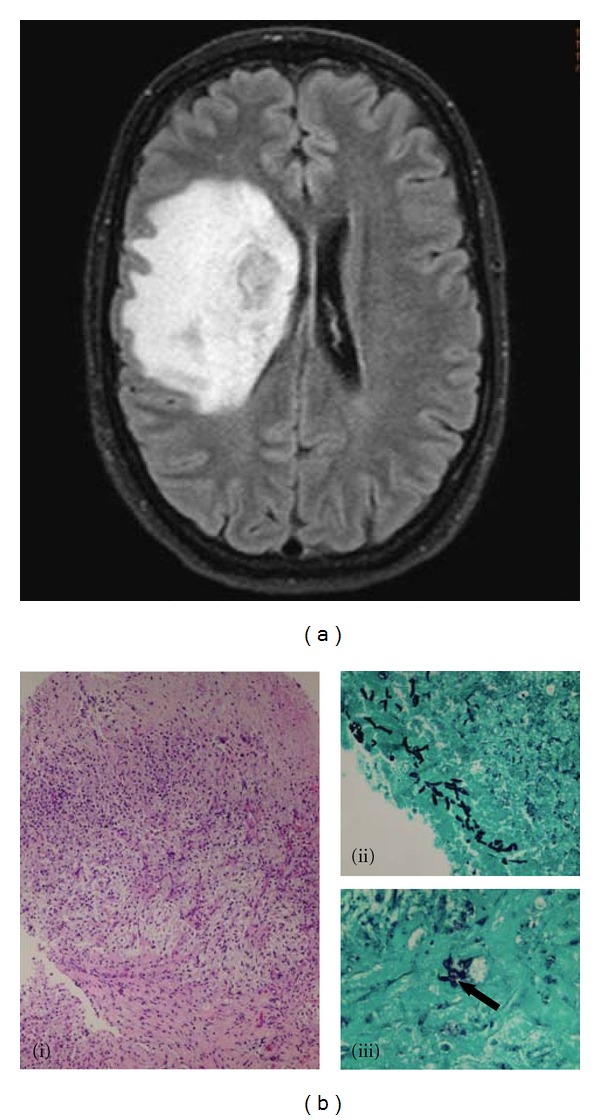
(a) FLAIR axial sequence of MRI of brain with gadolinium showing a large lesion in the right basal ganglia and frontal lobe with extensive edema and mass effect in keeping with a fungal abscess. (b) Brain biopsy (i). Representative area of the brain biopsy shows relatively acellular necrotic material in the top 1/5 of the image, the dense pink well-formed capsule, of a chronic abscess, occupying the bottom 1/3 of the image and a looser admixture of lymphocytes, macrophages, and new blood vessels in the center of the field. Hematoxylin and eosin, original magnification 100x (ii). The dark black structures forming a linear array running obliquely from top left to bottom center are fragments of fungi. Gomori Methenamine Silver, original magnification 400x (iii). The arrow indicates a single black fragment of a fungal structure with 45-degree branching, which is characteristic of aspergillus. Gomori Methenamine Silver, original magnification 600x.

Three weeks after discharge she presented to transplant clinic complaining of left-sided weakness and headache, in addition to dyspnea and cough. CT scan of her head showed extensive edema in the right cerebral hemisphere surrounding a mass-like lesion. Magnetic resonance imaging (MRI) reported this large lesion in the right basal ganglia, extending into the frontal lobe, suspicious for a fungal abscess (Figure 2(a)). She accordingly underwent stereotactic brain biopsy which showed a chronic abscess with fragments of fungal hyphae consistent with *Aspergillus *species (Figure 2(b)). Pertinent laboratory findings included elevated serum (1, 3)-beta-D-glucan at 376 pg/mL, and negative *toxoplasma gondii* PCR and galactomannan antigen. She was started on intravenous dexamethasone, and micafungin 100 mg/day was added to voriconazole. Her weakness and headache improved during the hospitalization and she was discharged home on a steroid taper, micafungin (to complete a four week course) and voriconazole.

Eight weeks later, after completing the course of micafungin, and resuming low-dose tacrolimus, a CT chest was done for persistent dyspnea and cough. It showed worsening opacities in bilateral lower lobes. Since clinical and radiographic findings were suggestive of ongoing aspergillosis, interferon gamma (IFN-*γ*) 200 mcg subcutaneously, three times a week was initiated and continued for six weeks. She was subsequently admitted and bronchoscopy performed. Bronchoalveolar lavage was positive for CMV and negative for *Aspergillus* species. Serum (1, 3)-beta-D-glucan level was 88 pg/mL and CMV PCR was 9650 cpy/mL. In addition to the IFN-*γ*, micafungin was restarted, and ganciclovir was added for her superimposed CMV pneumonitis and continued for three weeks. 

Surveillance CT scans of her head and chest were done one, three, four and seven months after beginning IFN-*γ*. Overall improvement was seen both clinically and radiographically and CMV PCR and (1, 3)-beta-D-glucan were negative on three separate measurements. However, CT scan of her chest at four months revealed an enlarging left upper lobe density which doubled in size at seven months. A CT-guided lung biopsy was done with cytology positive for non-small cell lung cancer. The current admission, one year after her initial presentation for pulmonary aspergillosis, was for a left upper lobectomy. The post-operative course was uneventful and her graft function remained stable with serum creatinine consistently between 1.3–1.5 mg/dL. Surgical pathology demonstrated moderately-differentiated adenocarcinoma with negative margins and lymph nodes. No fungal elements were seen on multiple sections. 

## 3. Discussion

We present a case of proven [9] invasive pulmonary and cerebral aspergillosis (and CMV pneumonitis), in a renal transplant recipient, treated successfully with adjunctive IFN-*γ* after combination antifungal therapy with voriconazole and micafungin that showed little clinical and radiographic improvement. Despite withdrawal or minimization of immunosuppression, renal function remained stable throughout one year. We submit that the course of IFN-*γ*, as described by others [8], eradicated the invasive fungal infection and in conjunction with treatment for CMV coinfection, was responsible for her symptom resolution. 

Initially recognized in patients with chronic granulomatous disease [10] or neutropenia, IA is now a well-recognized complication of solid organ transplantation (SOT), with an incidence of approximately 1% [1, 4]. Overall mortality in SOT is 41–76% and up to 90% in cerebral aspergillosis [1, 4, 11, 12]. Early diagnosis improves mortality [1], however is hampered by lack of reliable serum markers, and, at best, often only a probable diagnosis is reached, rather than proven [9]. Microbiologic evidence is rare, and sputum or bronchoalveolar lavage fluid culture cannot prove infection, as *Aspergillus* is ubiquitous. The galactomannan antigen assay has an overall sensitivity of 65–90% and a specificity of 89–98% but has primarily been studied in stem cell recipients and in hematologic malignancies [13–15] and recently was shown to be of lower yield in SOT and non-hematologic malignancies [16]. The beta-D-glucan assay appears to be more sensitive but must still be integrated with other clinical data, as it cannot differentiate between certain fungal infections, including candida and cryptococcus [13, 17]. The halo sign, a typical CT finding of IA, has been reported in 15–61% of patients; other possible radiographic findings include consolidations, cavitary lesions, and infarcts [16, 18]. Taken together, IA infection remains a lethal opportunistic infection following SOT and necessitates the integration of clinical, radiographic, microbiologic and immunologic data to effectively diagnose it.

Prior to the development of newer antifungal agents, amphotericin B was the primary therapy for invasive aspergillosis. Its use was limited by infusion reactions, nephrotoxicity and, electrolyte abnormalities and was associated with increased mortality compared with newer antifungals [1]. The introduction of voriconazole has improved survival with less toxic side effects when compared to amphotericin B [2], however mortality remains high. Since voriconazole has a strong inhibitory effect on cytochrome P450-3A4 activity, tacrolimus dosage must often be adjusted accordingly. Indeed in one case report, the potential nephrotoxic effect of this drug combination necessitated the discontinuation of tacrolimus [19]. Combination antifungal therapy has been attempted in efforts to improve outcomes; *in vitro* studies showed that for some isolates (primarily *Aspergillus fumigates*), the addition of micafungin to voriconazole raises the minimal inhibitory concentration (MIC) and enhances maximal killing effects of voriconazole against aspergillus hyphae [20]. This synergy was not seen in other species [21]. Importantly, the use of combination antifungal therapy was not associated with a mortality benefit in SOT patients with invasive aspergillosis [1]. Additionally, concern exists over increasing azole resistance; in one study of *Aspergillus fumigatus* isolates from 1997 to 2007, resistance to azoles increased from 0 to 17% over the ten-year period [3]. The relatively poor performance of current antifungals, as evident by high mortality rates, and rising azole resistance both necessitate a different approach to treatment of invasive fungal infections in immunocompromsied hosts. 

Early clinical experience with IFN-*γ* in treating refractory invasive fungal infections has demonstrated surprisingly good outcomes. To the best of our knowledge, there are 7 reported cases of renal transplant recipients with invasive fungal infections treated with IFN-*γ*, in conjunction with standard antifungal therapy, of which 3 had *Aspergillus fumigatus *[8] IFN-*γ* was administered for six weeks, with dramatic, immediate results and no relapse. Furthermore, allograft rejection, a distinct concern when using IFN-*γ* in transplant patients, was not encountered. Mechanistically, protection against IA infection in mice has been associated with increased levels of TNF-*α*, IL-2, and IFN-*γ* [6], Indeed, the phagocytic cells of mice resistant to aspergillus infection were better at eradicating fungal hyphae and conida while simultaneously producing more TNF-*α* and IFN-*γ* [7]. In healthy, immunocompetent humans, exposure to *Aspergillus fumigatus* caused the release of IFN-*γ*, demonstrating an intact Th1 response [22]. This protective effect of IFN-*γ* is explained by its immunologic action of stimulating B-cells, Th1 cells, neutrophils, and phagocytes. specifically, it promotes Th1 cytokine induction of TNF-*α*, causing the upregulation and activation of alveolar macrophages [23]. Current immunosuppressive protocols for SOT are potent inhibitors of these cell-mediated immune responses. Tacrolimus has been shown to inhibit intragraft mRNA expression of the Th1-cytokines IFN-*γ*, IL-12, and TNF-*α* [24], and in healthy human cells treated with tacrolimus and cyclosporine, a dose-dependent reduction in natural killer cell degranulation and IFN-*γ* production was seen [25]. 

Our case adds to the literature demonstrating successful and safe use of IFN-*γ* in IA in renal transplant recipients. We propose that IFN-*γ* be considered early in the course of treatment of invasive aspergillosis either in combination with other antifungals or alone. The enhancement of the immune system with immunomodulatory agents is an attractive alternative or adjuvant to established antifungal therapy, and has potential to impact morbidity and mortality. Their role in the treatment of invasive fungal infections is evolving.
